# Nanoparticle-Induced Complement Activation: Implications for Cancer Nanomedicine

**DOI:** 10.3389/fimmu.2020.603039

**Published:** 2021-01-08

**Authors:** Ninh M. La-Beck, Md. Rakibul Islam, Maciej M. Markiewski

**Affiliations:** ^1^ Department of Immunotherapeutics and Biotechnology, Jerry H. Hodge School of Pharmacy, Texas Tech University Health Sciences Center, Abilene, TX, United States; ^2^ Department of Pharmacy Practice, Jerry H. Hodge School of Pharmacy, Texas Tech University Health Sciences Center, Abilene, TX, United States

**Keywords:** nanomedicine, complement, activation, immunosuppression, tumor microenvironment, cancer, nanoparticle

## Abstract

Nanoparticle-based anticancer medications were first approved for cancer treatment almost 2 decades ago. Patients benefit from these approaches because of the targeted-drug delivery and reduced toxicity, however, like other therapies, adverse reactions often limit their use. These reactions are linked to the interactions of nanoparticles with the immune system, including the activation of complement. This activation can cause well-characterized acute inflammatory reactions mediated by complement effectors. However, the long-term implications of chronic complement activation on the efficacy of drugs carried by nanoparticles remain obscured. The recent discovery of protumor roles of complement raises the possibility that nanoparticle-induced complement activation may actually reduce antitumor efficacy of drugs carried by nanoparticles. We discuss here the initial evidence supporting this notion. Better understanding of the complex interactions between nanoparticles, complement, and the tumor microenvironment appears to be critical for development of nanoparticle-based anticancer therapies that are safer and more efficacious.

## Overview of Cancer Nanomedicine

Nanomedicine is a submicroscopic platform for effective and smart drug delivery, which enables direct drug interactions with cancer cells and their biological milieu. The main tool of this platform are nanoparticles, a heterogeneous group of engineered drug carriers, defined by a size within the nanometer scale, which includes: liposomes, polymer-conjugates, and micelles (1). The therapeutic potential of nanoparticles for cancer treatment lies in their ability to passively deliver drug to tumor tissue *via* the enhanced permeability and retention (EPR) effect (2). The EPR effect results from an increased vascular permeability of tumor blood vessels, which is linked to neoangiogenesis (3). Importantly, the size of nanoparticles enables their extravasation only in tumors but not in normal tissues. The nanoparticle formulation increases their half-life in circulation, leading to an increased number of passages of drug/carrier complex through the tumor vascular beds. The optimal size range to assure EPR effect appears to be between 20 and 200 nm (in approximate diameter). This ability of nanoparticles to specifically target tumors significantly attenuates drug toxicity. Additionally, the encapsulation of nanoparticles protects the drug from degradation (4). Several nanoparticle-based therapies have been approved because of improved efficacy and tolerability. The most common nanoparticles among the approved agents are liposomes, however, there are other nanoparticle-delivery platforms, including albumin-conjugated micelles and polyethylene glycol (PEG) conjugates (5) (**Table 1**). Additionally, a large repertoire of nanoparticle systems is under preclinical and early phase clinical development including biopolymers (chitosan, alginate, cellulose, hyaluronic acid), dendrimers, inorganic nanoparticles (Au, Ag, iron oxide, silica, etc.), quantum dots, and the combinations thereof (12–14). These novel nanoparticle systems are likely to become more clinically relevant as there is an increasing interest and research efforts focused on the integration of diagnostics and therapeutics within the cancer nanomedicine field (15, 16).

**Table 1:** Nanoparticle formulations used clinically for treatment of cancer.

Brand Name	Initial Approval	API	Platform	Indication
Oncaspar^®^ (6)	1994	Asparaginase	Polymeric Protein Conjugate	Acute lymphoblastic leukemia
Doxil^®^ (6) Lipodox^®^ (7)	1995	Doxorubicin	Pegylated liposome	Ovarian cancer, breast cancer,Kaposi’s sarcoma
DaunoXome^®^ (6)	1996*	Daunorubicin	Non-pegylated liposome	HIV-associated Kaposi’s sarcoma
Depocyt^®^ (6)	1999*	Cytarabine	Non-pegylated liposome	Lymphomatous meningitis
Myocet^®^ (8)	2000	Doxorubicin	Non-pegylated liposome	Metastatic breast cancer
Eligard^®^ (6)	2002	Leuprolide Acetate	PLG Polymer	Prostate cancer
Mepact^®^ (9)	2004	Mifamurtide	Non-pegylated liposome	Osteosarcoma
Abraxane^®^ (6)	2005	Paclitaxel	Albumin-conjugated micelle	Breast cancer
NanoTherm^®^ (10, 11)	2010	Iron Oxide	Iron oxide nanoparticle	Glioblastoma (thermo-ablative therapy)
Marqibo^®^ (6)	2012	Vincristine Sulfate	Non-pegylated liposome	Philadelphia chromosome negative acute lymphoblastic leukemia
Onivyde^®^ (6)	2015	Irinotecan	Pegylated (Stealth)	Pancreatic adenocarcinoma
Vyxeos^®^ (6)	2017	Cytarabine and Daunorubicin	Non-Pegylated	Therapy-relatedacute myeloid leukemia

*Discontinued and no longer in clinical use.

The transformation of a “free” drug, usually less than 1–2 nm size, into a nanoparticle, with ~1 million-fold greater volume and loaded with thousands of drug molecules, is an extraordinary pharmaceutical challenge with significant pharmacological and biological consequences. For example, unlike traditional small molecule drugs, nanoparticles have a tendency to interact with the innate immune system (17). The cells that primarily interact with systemically administered nanoparticles are mononuclear phagocytes such as tissue-resident macrophages, including hepatic Kupffer cells, and circulating monocytes. These interactions result in the clearance of nanoparticle-delivered drugs from the circulation and their sequestration in organs enriched in macrophages such as liver and spleen (18, 19). The nanoparticles also interact with plasma proteins like immunoglobulins, IgG and IgM, and complement proteins (20). These proteins adsorb to the surface of nanoparticles forming a protein corona (21, 22), which contributes to nanoparticle opsonization, phagocytic clearance, the formation of immune complexes, generation of immunogenic epitopes from self-antigens, and activation or suppression of the immune responses (21–23). The composition of the protein corona is dynamic, highly variable, and depends on the physicochemical characteristics of the nanoparticle and fluctuations in the host circulating proteins. The interactions of nanoparticles with circulating complement proteins leads to the activation of complement cascade (17, 24–26) and the subsequent generation of opsonins (e.g., C3b), anaphylatoxins (e.g., C3a and C5a), and C5b-9 complex, known as terminal complement complex (TCC) or membrane attack complex (MAC). The anaphylatoxins, especially C5a, are associated with acute infusion reactions in patients, known as complement activation-related pseudoallergy (CARPA) (27).

## Mechanisms of Nanoparticle-Induced Complement Activation

**Figure 1:**
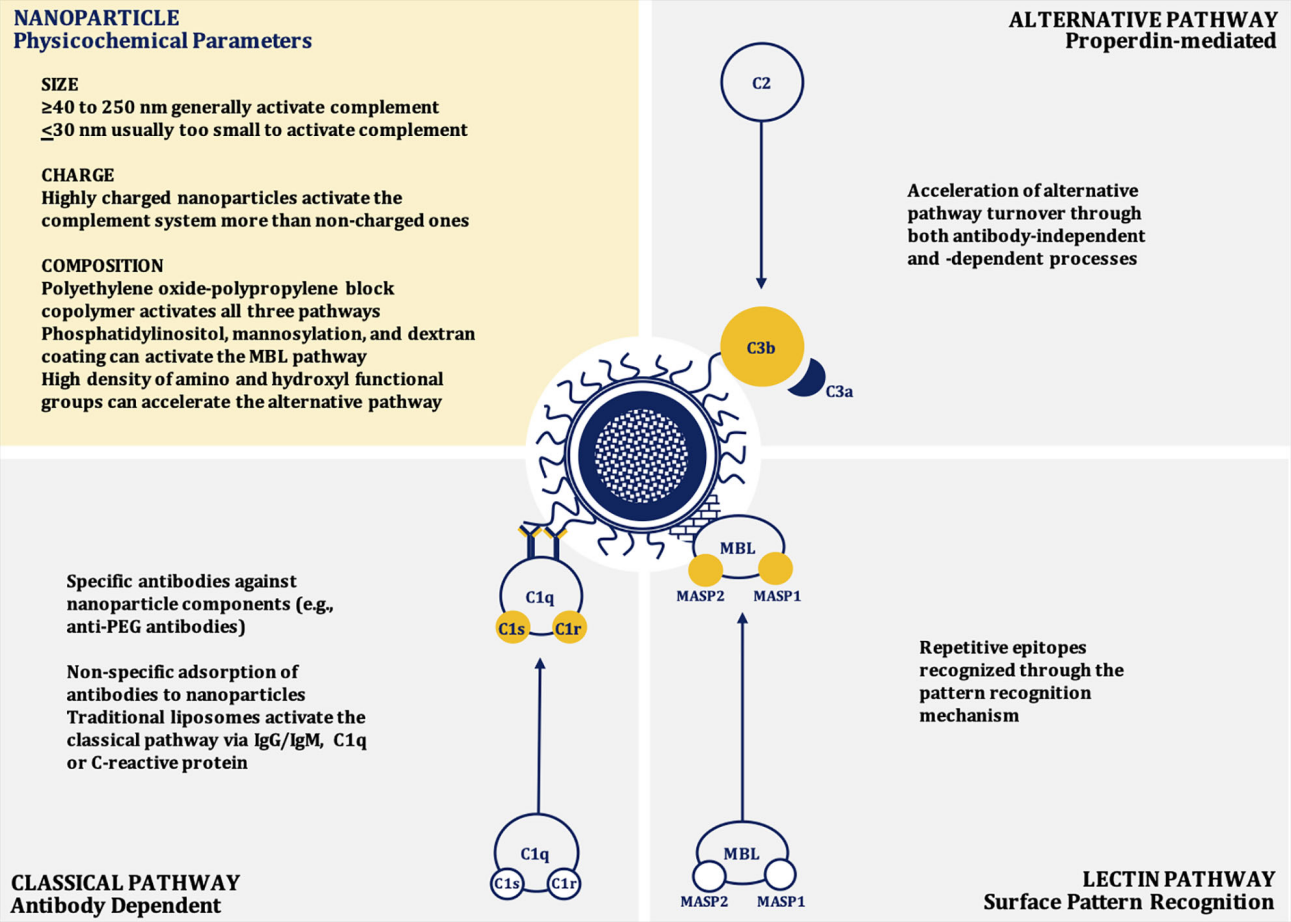
Mechanisms of nanoparticle-induced complement activation.

Nanoparticle-mediated complement activation is a multifaceted process that depends on the physicochemical characteristics of the nanoparticle including: surface chemistry and topography, charge (zeta potential), size, and shape (28–38). Depending on the composition, nanoparticles may induce complement activation through the classical (IgG/IgM/C-reactive protein-mediated), mannose-binding lectin (MBL), or alternative (properdin-mediated) pathways, or any combination of these canonical pathways (39–44) (**Figure 1**).

### Size and Shape

In general, as size increases, nanoparticles induce greater complement activation and are also more likely to be internalized by phagocytic cells, presumably due to enhanced opsonization by complement proteins (45, 46). Nanoparticles between 40 to 250 nm in size induce a potent activation of the complement system through the classical pathway similar to dextran coated nanoparticles with a size of ~250 nm (33). However, if the size of the particle is very large (~600 nm diameter), the activation of complement is reduced when adjusted for surface area (38, 46). Dextran-coated superparamagnetic iron oxide (SPIO) core-shell nanoworms of a size of ~200 nm are opsonized by C3b.This C3b engages with properdin to recruit more C3b to form C3bBb, the C3 convertase of the alternative pathway (47, 48). When the size of the nanoparticle is at or below 30 nm diameter, they are usually too small to efficiently trigger the calcium-dependent complement activation pathways, such as the classical or lectin pathways, due to the relatively large size of C3b. The complement opsonin C3b occupies an area of about 40 nm^2^, therefore, very small nanoparticles do not have enough surface area to adsorb C3b molecules. Consequently, most of the C3 cleavage fragments will be released rather than be deposited on to the surface of these nanoparticles (38, 46, 49).

In addition to size, the particle shape and curvature also play roles in complement activation. Studies with SiO_2_ nanoparticles of different sizes (8, 32, and 68 nm) demonstrate that surfaces with the sharp curvature can reduce complement activation. Peptidoglycan particles with a diameter of 50–100 nm and curvature in the range of 0.02–0.04 nm^−1^ induce stronger complement activation when compared to particles with shallower or sharper curvatures. If the curvature is sharper or shallower than 0.02–0.04 nm^−1^, the conformation requirement for complement activation through IgM and C1q is not optimal and leads to poor induction of the complement cascade (38, 50). Prolate ellipsoidal (rod) and oblate ellipsoidal (disk) shaped carboxylated polystyrene nanoparticles induce more profound and robust activation of the complement system than spherical nanoparticles when tested in porcine blood. However, when tested in human blood, the difference in complement activation between nanoparticles with different shapes were negligible (51). This phenomenon is also seen with spherical gold nanoparticles, gold nanorods, and gold nanostars (52). More research is needed to understand the mechanisms for this difference of complement activation between species.

### Composition, Surface Charge, and Zeta Potential

While nanoparticles less than 30 nm are less likely to induce the activation of the complement system, their composition also affects this process because of the interaction of a particular material with the surrounding biological milieu. Polyethylene oxide-polypropylene block copolymer poloxamer 407 micelles of ~25 nm significantly activate the complement system *via* all three canonical pathways, while similar sized PEG-phospholipid micelles fail to activate the complement system (46, 53, 54). The closer inspection revealed that the poloxamer 407 component leads to the generation of larger particles with a range of 100 nm to nearly 1 µm in human plasma. Likely these particles interact with chylomicrons and other lipoprotein classes to form large aggregates that lead to the more potent activation of the complement system (54).

The surface charge of nanoparticles also impacts their interaction with complement. Nanoparticles with anionic surfaces (e.g., liposomes) attract Ca^2+^ ions that are vital for the activation of complement system through the classical pathway (28, 55, 56). These anionic charges derive from cardiolipin, phosphatidylserine, phosphatidic acid, and phosphatidylglycerol incorporated within the structure of liposomes. The complement protein C1q can also directly bind to these anions through hydrophobic interactions and/or hydrogen bonding (57, 58). Cationic or positively charged liposomes containing lipids, including stearylamine or 1,2-bis(oleoyloxy)-3-(trirnethylammonio) propane, activate the complement system by interacting with proteins of the alternative pathway. Neutral liposomes poorly interact with the complement components and poorly activate the complement system (28, 56, 59). Consequently, nanoparticles containing polypropylene sulfide, lipid nanocapsules, polycations, polyplexes, and polystyrene that are highly charged are more potent activators of complement than particles with low or no charge (60–63). The important role of surface chemistry in complement activation is obvious when nanoparticles are coated with surface-charge-neutralizing polymers, such as polyethylene glycol (PEG). This coating leads to a reduction in nanoparticle-mediated complement activation (60). Suppression of complement activation by neutralizing polymers on the surface of anionic nanoparticles can occur even if the net charge remains slightly negative. For example reducing the net charge from −27.17 to −6.046 mV was sufficient to mitigate nanoparticle-induced complement activation (64).

Lipid bearing nanoparticles (e.g., liposomes) can activate the classical complement pathway *via* interactions between IgG and/or IgM and the phospholipid head-groups and cholesterol components (43, 57, 65, 66). Anti-phospholipid antibodies can also bind to other suitable epitopes found on the liposome surface, such as apolipoprotein H (66, 67). However, there is significant inter-individual variability in the specificity of these antibodies, which may contribute to heterogeneity in nanoparticle-induced complement activation in patients (65, 66). The classical pathway can also be activated by liposomes through the adsorption of C-Reactive Protein (CRP) to the liposomal surface. This CRP subsequently interacts with C1q (66, 68, 69). Liposomes containing phosphatidylinositol may also trigger the lectin pathway through binding to MBL. This initial event leads to MBL-Associated Serine Protease-2 (MASP-2) activation, triggering the complement cascade (40, 66). Similar mechanisms appear to be applicable to mannosylated liposomes. Liposomes can also contribute to the alternative pathway through antibody-independent direct C3 adsorption and C3 conformational changes that lead to the generation of structures resembling C3b and subsequent formation of the alternative pathway C3 (C3bBb) convertase. The alternative pathway is also triggered when the C3 binds to the Fab portion of liposome-bound antibodies (41, 42, 66, 70). In addition to charge neutralization, incorporating mPEG may reduce or delay complement activation through steric hinderance preventing interactions with blood proteins (39, 53). A high density of polymeric chains may lead to the compression of the chains on the surface of the nanoparticle. Whereas a low density may lead to interpenetration of protein molecules on the surface of the mPEG coated nanoparticles. Compression of the polymeric chains may cause steric hindrance that results in reduced protein interactions, consequently, the reduction in complement activation (71). When the surfaces of nanoparticles are modified, however, this can lead to different conformations of nanoparticle surface architectures that can have varying effect on the complement system. If the said surface is modified with mPEG, for example, different conformations can be generated, such as “mushroom,” “mushroom-brush,” or “brush.” Changing the surface conformation from “mushroom”-like to the other conformations leads to a reduced complement activation and shifts the pathway from the classical to the lectin (35).

### Topography and Surface Chemistry

Nanoparticle surfaces that have repetitive epitopes may trigger the activation of the complement system through the pattern recognition mechanisms, which are dependent on the surface topography. For example, the nanoparticles coated with star-shaped polyethylene oxide-polypropylene block copolymer (poloxamine 908) have the repetitive patterns of polarity and hydrophobicity. These patterns can be manipulated by altering the density of poloxamine on the nanoparticle surface, and, therefore, the docking sites for complement pattern recognition receptors can be altered (35, 46, 72).

A high density of amino and hydroxyl functional groups on the surfaces of nanoparticles can induce a nucleophilic attack by these chemical moieties on the internal thioester bond within the α-chain of nascent C3b, resulting in the acceleration of alternative complement activation pathway (46, 73, 74). Additionally, nanoparticles with surface polysaccharides that are cross-linked facilitate the activation of the complement system. This activation is partially inhibited if the hydroxyl groups are substituted with carboxymethyl groups (75, 76). The impact of the surface chemistry on the activation of the complement system becomes very complex when the interspecies variation is considered. For example, superparamagnetic iron oxide (SPIO) nanoparticles coated with dextran activate the classical complement pathway in mice, but when tested in human serum, the alternative pathway was found to be activated (76–80).

### Drug Payload

The composition of the nanoparticle carrier clearly plays a role in complement activation, however, the influence of the payload (i.e. encapsulated or conjugated drug molecules) on the complement system has not been thoroughly studied. Doxil^®^, a PEGylated liposomal doxorubicin (PLD), activates the complement system more than liposomes of similar size and formulations that do not contain doxorubicin (46, 81). One of the characteristic features of PLD liposomes is their oblate/disc shape, whereas placebo liposomes are spherical. The oblate shape of PLD is due to deformation of the liposomes by crystalized doxorubicin that is loaded into these liposomes. PLD may trigger more complement activation *via* classical and/or alternative pathways, partly due to the altered phospholipid arrangement and partly due to the surface presence of doxorubicin crystals. It has also been observed that the administration of placebo PEGylated liposomes in rats induced an IgM-mediated complement activation, which increased the hepatic clearance of the second dose of the liposome. In the case of the doxorubicin encapsulated PEGylated liposomes, however, the second dose showed similar long-circulating half-life. This phenomenon is believed to be related to the action of doxorubicin, which can kill B cells, responsible for producing IgM (26, 46, 82, 83).

## Clinical Impact of Nanoparticle-Induced Complement Activation

Complement activation triggered by nanoparticles results in both the liberation of proinflammatory mediators such as anaphylatoxins and the opsonization of nanoparticles with C3b, which interacts with phagocytes (84). The anaphylatoxins (C3a, C4a, and C5a) stimulate the release of additional inflammatory mediators (e.g. histamine) by the immune cells. This sequence of inflammatory events was observed in connection with CARPA reactions in porcine and canine models (85). Several formulations of nanoparticles in clinical use (Doxil^®^/PLD, DaunoXome^®^, AmBisome^®^, Abelcet^®^, Amphocil^®^) have been shown to cause hypersensitivity reactions in patients that are consistent with CARPA (86). After intravenous administration, PLD activates complement in the blood of cancer patients, and the extent of complement activation (as measured by formation of s5b-9) correlated with the development of acute infusion reactions (27). Although complement activation induced by nanoparticles is well established (86), the clinical occurrence of CARPA does not appear to be as prevalent as would be expected from *in vitro* studies. For example, PLD induces significant complement activation *in vitro*, however, the occurrence of acute infusion reactions in patients is typically less than 10% and can be mitigated with premedications and by slowing the rate of infusion (27). Nonetheless, undesired interactions with circulating complement proteins can affect the pharmacokinetics and tolerability of nanoparticle-mediated drugs.

Coating nanoparticles with polyethylene glycol (“pegylation”) has become widely used to reduce complement activation, improve stability in plasma, and prolong circulation time, which are all important for effective tumor targeting (87, 88). However, these approaches do not entirely abolish the immune reactions to nanoparticles (39). Several groups have demonstrated that the initial systemic administration of pegylated nanoparticles induces production of anti-PEG IgM antibodies that enhance immune recognition and clearance of the second dose of nanoparticles in preclinical models. Of note, this “accelerated blood clearance” (ABC) phenomenon has not been reported in patients, and its clinical relevance is currently unclear. In fact, the opposite has been observed in patients treated with PLD, where clearance rates decreased with repeat administration, up to 30% by the third cycle (89).

Nanoparticle-induced complement activation is generally perceived as undesirable when nanoparticles administered systemically lead to complement-mediated infusion reactions (27, 90). While uncontrolled complement activation can induce inflammatory and life-threatening consequences, controlled complement activation by nanoparticles may be beneficial for vaccination strategies (91–93). Opsonization of pathogens by complement proteins facilitates their uptake by antigen presenting cells *via* complement receptors CD21 and CD33 (94). In the case of nanoparticle-based vaccines, particle-induced complement activation products can act as endogenous vaccine adjuvants to enhance antigen uptake and recognition by antigen-presenting cells. Production of the complement cleavage products C5a and C3a locally at the APC and T cell interface is important for T cell costimulation and survival (95). Antigens that are opsonized by complement C3d engage both B cell receptors and the complement CD21 costimulatory receptor, activating antibody responses more efficiently (96, 97). These complement components can be leveraged by nanoparticle vaccines, where the localized activation of the complement system enhances the immune response against the nanoparticle-delivered antigens (98). Thus, the propensity of nanoparticles to induce complement activation can theoretically be leveraged to facilitate their efficacy as antigen carriers for vaccinations (98, 99).

Several studies of the last decade clearly demonstrated that complement proteins and complement activation accelerates tumor growth in mouse models and patients. Therefore, given the propensity of nanoparticles that are administrated often to cancer patients to activate complement, it is conceivable that this activation may have also reduced therapeutic efficacy of nanoparticles-based drugs. We will explore this possibility through the remaining sections of this review.

## The Role of Complement in Cancer-Associated Immune Dysfunction, Angiogenesis, and Metastasis

The role of the complement system in cancer has been implicated for decades. The early studies demonstrated that several complement proteins are expressed or deposited in common human solid tumors (100). Given a well-established role of complement in innate immunity and in the initiation and propagation of the subsequent adaptive immune responses against microbial pathogens, these findings were thought to support the theory that complement also contributes to antitumor immune responses and tumor immune surveillance (100). This notion appears to be strengthened by a significant role of complement and complement-dependent cytotoxicity (CDC) in killing tumor cells by therapeutic monoclonal antibodies (101). However, the studies of the last decade clearly indicate that complement proteins and complement activation, in the absence of therapeutic antibodies, promote tumor growth in mouse models and cancer patients (102). The original discovery of tumor-promoting roles of complement linked these complement functions to the complement anaphylatoxin C5a receptor 1 (C5aR1)-dependent regulation of myeloid-derived suppressor cells (MDSC) and their C5aR1-dependent recruitment to tumors (103). MDSC have recently emerged as one of the most important cell subsets in the tumor microenvironment (TME), responsible for suppression of antitumor T cell-responses, enhancement of tumor-angiogenesis, and resistance to therapy (104). In fact, C5aR1-dependent regulations of MDSC led to the suppression of antitumor CD8^+^T cells because depletion of these cells by anti-CD8 neutralizing antibody erased beneficial effects of C5aR1 blockade on tumor growth in a mouse model of HPV-induced cancer (103). This study has linked the complement activation in tumors to the classically pathway, as C3 cleavage fragments colocalized with C1q in tumors. C1q initiates the classical pathway (105). In addition, mice deficient in complement fragments C4, which is required for the classical and lectin pathways to progress, had reduced tumor growth. Conversely, mice deficient in factor B, a key protein of the alternative pathway grew tumors in a similar rate as wild type littermate controls (103). Several follow-up studies have confirmed these initial findings in different mouse models (106) and discovered other complement-mediated mechanisms contributing to immune suppression in TME (102).

MDSC also play an important role in inducing another immunosuppressive subset-T regulatory cells (Tregs). Consistent with this MDSC function, the reduced number of Tregs were found in blood and the lungs of C5aR1-deficient mice in a model of metastatic breast cancer (107). The mechanisms of C5aR1-mediated induction of Tregs were connected to the regulation of TGF-β1 and IL-10 in cells of myeloid-origin in the lungs (107). TGF-β1 and IL-10 secreted from myeloid cells have been previously implicated in generation of Tregs in tumors (108). Upon C5aR1 inhibition, the reduced numbers of Tregs in the lungs correlated with the reduced lung metastatic burden (107). C5aR1 signaling is also implicated in generation of Tregs in tumors in a transgenic Her2/neu-driven model of breast cancer (109). The reduced generation of Tregs when C5aR1 signaling was blocked, with a specific C5aR1 inhibitor (PMX53) (110), was caused by the decreased production of TGF-β1 and increased expression of IL-6 in myeloid cells in tumor infiltrating lymph nodes (109), as the interplay between these two cytokines is pivotal for generating various subsets of T cell effectors (111).

In addition to regulating myeloid-origin cells such as MDSC and tumor-associated macrophages (TAM) (112), C5aR1 and the complement anaphylatoxin C3a receptor (C3aR) synergistically impair cytolytic activity of tumor infiltrating CD8^+^T cells (TIL) by inhibiting expression of IL-10 in these cells (113). C3, required for complement activation and generation of the complement anaphylatoxins C3a and C5a, was shown to be produced by TIL. Through their reciprocal receptors expressed in TIL, C3a and C5a blocked IL-10 expression in these cells in autocrine manner (113). The expression of C3aR and C5aR1 in TIL indicates that TME favors expression of these receptors in T cells because non-activated T cells in the blood, spleen, or lymphoid organs were repeatedly shown to lack C3aR and C5aR1 protein (114).

Interestingly, complement appears to promote tumor angiogenesis, as indicated by reduced vascular density and impairment of endothelial cell function in C3aR-and C5aR1-deficient mice in a transgenic model of ovarian cancer (115). C1q, which initiates the classical complement pathway of activation, was found in stroma and vasculature of several human cancers, and C1q-deficient mice exhibited reduced vascular density in tumors in a B16 melanoma model (116). The most recent study, showing the striking impact of complement genes on outcomes in renal cell carcinoma (RCC), found that C3aR-deficiency or blockade and C5aR1 blockade were all associated with reduced vascular density of tumors in a mouse model of RCC (117). Similar to the first study reporting tumor promoting role of complement (103), this recent work linked the activation of complement in a mouse model to the classical pathway. Interestingly, the comprehensive analysis of TME transcriptome of tumors from RCC patients revealed a significant association of complement genes including C1q signature with highly aggressive inflammatory subtype of RCC (117). Consistent with these finding, another report on a role of complement in RCC found associations of genes encoding early complement fragments, involved in the classical pathway, with poor prognosis (118).

Finally, complement promotes tumor growth through autocrine signaling in tumor cells, and this effect was independent of TIL in a model of ovarian carcinoma. C5aR1 and C3aR signal through the PI3K/AKT pathway, and silencing the *PI3K* or *AKT* gene in tumor cells reduced impact of C5aR1 and C3aR stimulation on tumor growth. In patients with ovarian or lung cancer, higher C3 or C5aR mRNA levels in tumors were associated with decreased overall survival (119). These studies together support a key role of complement system in regulating TME, however, they mainly focused on growth of tumors in primary sites. Several recent comprehensive review articles cover this topic in detail (102, 120).

Recent work extends the findings on TME to the metastasis promoting functions of complement. The C5a/C5aR1 regulatory axis was demonstrated to recruit MDSC to the lung and liver premetastatic niches in a model of metastatic breast cancer (107). This recruitment and activation of MDSC resulted in the reduced infiltration of these organs by CD8^+^T cells that appears to eliminate metastasizing tumor cells in these sites, as the depletion of CD8^+^T cells eliminated the beneficial effect of C5aR1 blockade on lung metastatic burden. Furthermore, impact of C5aR1 on antitumor immunity in metastatic sites was linked to Th2-oriented responses that rendered CD8^+^T cells dysfunctional (107). In addition to recruiting lung infiltrating MDSC, C5aR1 appears to be involved in regulating self-renewal of tissue-resident pulmonary alveolar macrophages (AM) in the lung premetastatic niche that, like MDSC, inhibit antitumor T cell responses by favoring generation of Th2 cells. In addition, AM reduced the number and maturation of lung dendritic cells by regulating TGF-β1 in the lung environment (121). Similar to findings from primary tumor sites (103, 117, 118), complement activation in the premetastatic niches seems to be associated with C1q-deposition and the classically pathway (122). C1q was demonstrated to bind to IgM-deposited in the premetastatic niche. These IgM likely belong to natural IgM that bind dying or damaged cells as demonstrated by colocalization of Annexin V (binding to apoptotic cells) with IgM fluorescence in the lungs prior metastasis (122).

In summary complement activation and generation of complement effectors seem to be pivotal for protumor complement functions. Several studies linked mechanistically activation of the complement cascade in tumors to the classical pathway. However, the alternative pathway is known to contribute to 80% of C5a generation when the complement cascade is activated through the classical pathway (123). Therefore, the alternative pathway amplification loop is very likely to contribute to complement activation in cancer. Of course, which mechanism is pivotal for complement activation is expected to tumor type-dependent. Finally, some complement functions do not require the activation of complement cascade. For example a proangiogenic role of C1q is not associated with the classical pathway but seems to involve the direct interaction of a globular C1q head with C1q receptors expressed on endothelial cells (116).

## Implications of nanoparticle drug delivery-induced inflammation for cancer

**Figure 2:**
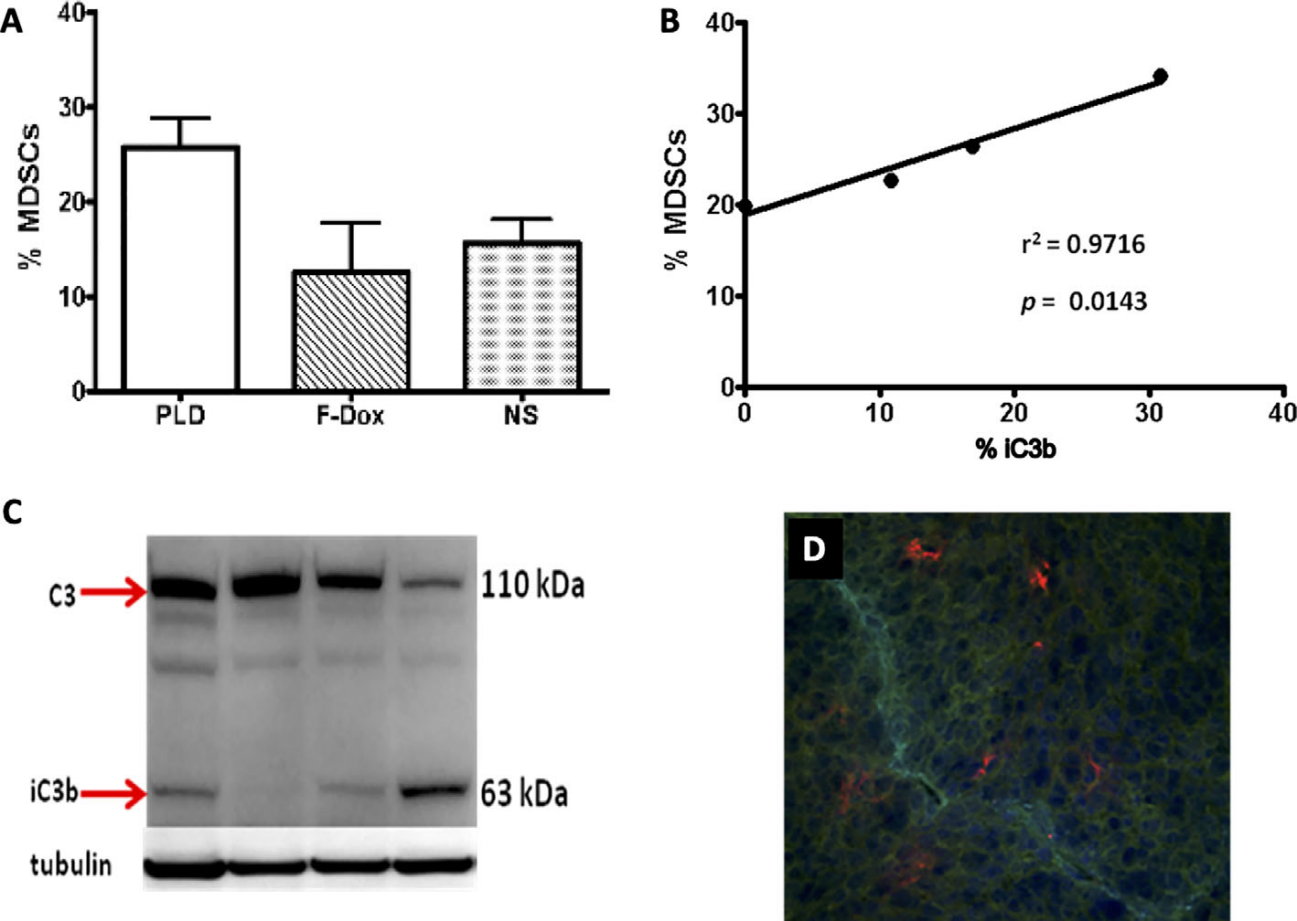
Liposome-associated complement activation in tumor tissue correlates with myeloid-derived suppressor cell infiltration. **(A)** Pegylated liposomal doxorubicin (PLD) treatment increased MDSC in tumors (p = 0.03) whereas free doxorubicin (F-Dox) showed no change in %MDSC (p > 0.05). Treatments were intravenously administered in C57Bl6 mice bearing TC-1 tumors implanted on the hind flank: PLD (n = 4), F-Dox (n = 4), and saline (NS; n = 5). **(B)** Increasing levels of complement activation in tumor correlates with increased tumor MDSC in PLD treated mice. **(C)** Complement activation was quantified using immunoblot analysis of tumor lysate and verified by immunohistochemical analysis of frozen tumor sections. **(D)** A representative image is shown; red = iC3b, aqua = CD31 (vasculature), blue = DAPI (cell nuclei).

Complement cleavage products and the uptake of nanoparticles by immune cells mediated by complement receptors may induce chronic inflammatory responses that could potentially negate the therapeutic effect of the payload. Indeed, there is an increasing evidence that nanoparticles could promote tumor growth in mice (124, 125). Polymer nanoparticles that are able to activate the complement system were found to increase tumor growth in a C5aR1-dependent manner, presumably through the liberation of C5a and the recruitment and activation of proinflammatory macrophages and Tregs (103, 126). We have also found that systemic administration of PLD to mice was associated with the increased infiltration of tumors by MDSC and the deposition of the complement cleavage products in tumors (**Figure 2**). Recently, we tested this pegylated liposomal carrier without any drug payload, and observed the significantly enhanced tumor growth in a syngeneic HPV-induced mouse tumor model (125). This enhanced tumor growth was associated with the suppression of antitumor immunity, indicated by blunting the production of cytokines in TAM and CD8^+^ T cells and the suppression of tumor antigen-specific immune responses. Moreover, tumor vascular density was significantly increased in mice receiving pegylated liposomes, suggesting enhanced angiogenesis. Mechanistically, *in vivo* treatment with liposomes increased expression of arginase-1 (typical of M2 macrophages and MDSC) associated with the accumulation of TAMs with a mixed M1/M2 phenotype when compared to vehicle treated mice that had predominantly M1 macrophages in tumors (127). These findings suggest that nanoparticle-induced immune modulation may theoretically attenuate therapeutic efficacy of nano-encapsulated drugs (120, 128–130). This may be especially relevant for cancer patients as a result of profound and heterogenous immune dysfunction (131).

While complement activation associated with these “placebo” nanoparticles have the potential to promote tumor growth, this effect was not associated with drug-loaded nanoparticles. It is likely that the anticancer drug, loaded within the nanoparticle, mitigates the harmful carrier-related effects by inhibiting both tumor cells and TAMs that internalize the nanoparticles. Thus, for cytotoxic chemotherapies, the tumor-enhancing potential of nanoparticle-induced complement activation may not be fully appreciated. However, when nanoparticles are used for delivery of other drugs including immunotherapeutics, which do not act *via* direct tumor cell killing, nanoparticle-induced complement activation could conceivably diminish their efficacy. Another consideration is that complement activation in the blood, which occurs after intravenous infusion of nanoparticle drugs, is transient and likely do not persist long enough to impact tumor growth in the long term. Nonetheless, chronic inflammation and complement activation can promote tumor progression, although it has not been determined whether nanoparticles that accumulate in the tumor tissue induce chronic complement activation.

The major pitfall in the studies of the complex interactions between nanoparticles and the innate immunity is that *in vitro* studies and studies in “healthy” animals do not sufficiently mirror the biological interactions of nanomedicines with the immunity of cancer patients. The xenograft tumor models are the predominant *in vivo* models used to demonstrate anticancer efficacy of drugs including nanomedicines. However, they rely on immunodeficient mice. The genetically engineered and syngeneic tumor models that utilize immunocompetent mice would be better options for assessing the complex interplay between the tumor immunologic milieu and nanomedicine. The selection of animal species for use in preclinical tests of nanomedicines also has a major impact on the preclinical toxicology results. Some conventional preclinical models (rats and non-human primates) may be insensitive to complement activation and cytokine-storm induction by nanoparticles (132–137). In such cases, supplementing *in vivo* studies with *in vitro* assays utilizing human blood should be considered. Another consideration for selecting an animal model is related to the variable sensitivity of animal strains to a particular type of immunotoxicity. For example, rabbits are more sensitive to cytokine and complement-mediated toxicities than rodents. Among rodents, strains may differ in their selectivity to nanoparticle clearance. For example, Balb/c and C57BL/6 mice commonly used in preclinical studies demonstrate a different pattern of nanoparticle uptake due to their Th2 and Th1 bias, respectively (138).

## Conclusions

Over two decades after the approval of the first nanoparticle-mediated anticancer drug, there has yet to be a major shift in cancer treatment paradigms linked to nanoparticles, contrary to what was expected based on preclinical studies of cancer nanomedicines (130). Only two anticancer nanoparticles are used as front-line therapies: nanoparticle albumin-bound paclitaxel (*nab*-paclitaxel; Abraxane^®^) for advanced non-small cell lung cancer and metastatic pancreatic adenocarcinoma, and liposomal daunorubicin cytarabine (CPX-351; Vyxeos^®^) for treatment-related acute myeloid leukemia and acute myeloid leukemia with myelodysplastic changes. Nonetheless, nanoparticle-mediated drug delivery is a proven strategy to mitigate toxicity of anticancer drugs in patients (139–141). The future of cancer nanomedicine is promising as recent new insights in understanding the role of the complement system in cancer will perhaps facilitate our understanding of how nanoparticle interactions with the innate immune system impacts drug pharmacology. If this knowledge gap can be addressed, it will lay the foundation for future work that will uncover the full clinical potential of cancer nanomedicines (18).

## Author Contributions

All authors listed have made a substantial, direct, and intellectual contribution to the work and approved it for publication.

## Funding

This work was funded by the National Institute of Health (RO1CA190209 to MM).

## Conflict of Interest

The authors declare that the research was conducted in the absence of any commercial or financial relationships that could be construed as a potential conflict of interest.
